# The Social Construction of a Concept—Orthorexia Nervosa: Morality Narratives and Psycho-Politics

**DOI:** 10.1177/1049732320911364

**Published:** 2020-05-18

**Authors:** Alison Fixsen, Anna Cheshire, Michelle Berry

**Affiliations:** 1 University of Westminster, London, United Kingdom

**Keywords:** eating disorders, social constructionism, orthorexia nervosa, health and wellbeing, Internet support, qualitative, triangulation, UK, USA

## Abstract

Our article explores orthorexia nervosa (ON)—an extreme fixation with healthy eating—from a social construction perspective. Interviews with people self-identified as “obsessed” with healthy eating or having ON (“Identifiers”) and nonmedical professionals working with ON (“Professionals”) were comparatively analyzed, along with orthorexia threads from an eating disorder website (“Posters”). Participants made sense of and rationalized their attitudes and feelings concerning healthy eating and aligned themselves according to their interests. Identifiers and Posters applauded “healthy eating” and regarded consumption of “impure” foods as leading to ill-health. Some framed their dietary discipline within an ethically motivated lifestyle, while others were preoccupied with appearance or weight management. Professionals expressed concern for, and disapproval of, extreme views and behaviors in clients and parental and social influences supporting them. Debates surrounding orthorexic practices are tangled; some individuals need help, yet dangers lie in over medicalizing or “troubling” what may be a preferred lifestyle.

## Introduction

The term orthorexia nervosa (ON) describes an eating disorder (ED) in which an excessive fixation on the consumption of healthy food and its biological purity leads to a restrictive diet, ritualized patterns of eating, and the rigid avoidance of foods considered unhealthy or impure (Koven & Wabry, 2015). Those with other EDs, women, adolescents, and sportspeople are considered at higher risk of developing ON (Bartrina, 2007; Håman et al., 2017; Koven & Wabry, 2015). Although qualitative studies of obsessive healthy eating practices do exist (Musolino et al., 2015), studies of orthorexia have largely been aimed at refining individual clinical symptom pictures, often neglecting the role played by social elements such as culture, family, peer groups, and practitioners in this process (Duran et al., 2016). The social constructionist approach on the other hand takes a broad perspective of a dynamic society, regarding both the labels and meanings that individuals and institutions use for things as subject to constant reconstitution (Gergen, 1985). A social constructionist approach, for example, would seek to explain the framing of eating practices by both medical and nonmedical actors within a cultural paradigm. In this article, we use this approach to illuminate three different perspectives on extreme healthy eating: those who self-identify as highly preoccupied with healthy eating, professionals with expertise in ON, and posters on an ED social media site. By focusing on and comparing the narratives of individuals who differentially position themselves around debates concerning health and eating, we can begin to understand the social and psycho-political tensions around labeling healthy eaters as “troubled persons” (Gusfield, 1989). We begin with a brief history of extreme alimentary practices and their evolution into psycho-pathologies (De Vos, 2012).

### A Disordered History

The point at which an eating behavior becomes disordered is contentious; no more so than in the adoption of behaviors considered by their adherents to be health promoting. Health and humanitarian approaches have long promoted abstinence (e.g., fasting, avoiding meat, flesh, rich or refined foods) as a means of physical, mental, and spiritual purification. More recently, the term “clean eating” has been adopted to describe an approach to eating in which processed foods, including white sugar and processed meats, are eliminated (Allen et al., 2018). At the same time, a vast body of modern literature warns against the adoption of extreme eating patterns due to both their potential for pathological, even fatal, consequences for individuals (Arcelus et al., 2011) and their financial and psychological costs to society (Nagl et al., 2016). From a social normative perspective, EDs such as anorexia nervosa (rigid restriction of food intake) or bulimia nervosa (overindulgence followed by purging through induced vomiting or use of laxatives) can be framed as deviant, in that they violate social and cultural norms of self-preservation and eating behaviors (Taub, 2011). They may also be labeled as clinically deviant, with the patient denying their symptoms and showing little or no motivation to return to relative normality (Halmi, 2013). With the advent of pro-anorexia and similar ED forums, some have sought to resist stigmatization (Yeshua-Katz, 2015), but in doing so may be seen as posing a threat to public health (Christodoulou, 2012).

### The Construction of ON

Although the aberrant responses of patients with EDs to treatment have been widely studied (Faija et al., 2017; Musolino et al., 2015), the emotional responses of professionals to the disorders they treat are rarely scrutinized in any literature (Fixsen & Ridge, 2012). In a review of treatment resistant features in ED psychopathology, Halmi (2013) writes of an “omnipresent frustration in eating disorders” (p. 1), indicating an uncharacteristic sense of powerlessness on the part of professionals attempting to engage with, or to change the thought patterns and behaviors of, patients or clients with EDs. Such was the experience of Steve Bratman who, when working as a doctor of alternative medicine in mid-1990s America, encountered a high number of “health junkies” (similar to his former self) whose excessive focus and rigid adherence to their diets was accompanied by an attitude of self-righteousness, self-punishment, and social isolation (Bratman & Knight, 2000). Subsequently, Bratman coined the term ON from the Greek “orthos” (straight, proper) and “orexis” (appetite) for this phenomenon and it is from there that the formal orthorexia story begins. Bratman has since made it clear that he does not consider restricted dietary practices, such as veganism or following the “paleo” diet, to be pathological or deviant in themselves, but become so when obsessive thinking and self-punishment come into play, resulting in malnutrition and impaired daily functioning (Bratman, 2017). The perils and glamor of orthorexia have subsequently attracted intense media interest (“Is Orthorexia Nervosa the Eating Disorder for the Digital Age?,” 2019, Oksman, 2015), with the term verging on becoming common parlance.

As ON has found its way into the cultural vocabulary, the status of ON as clinically unique disorder rather than a variant of other disorders has become a hot topic of debate among clinicians and health researchers across the globe (Gramaglia et al., 2017; Koven & Wabry, 2015). Widely regarded as sharing traits with anorexia nervosa (Musolino et al., 2015) and bulimia nervosa (Dell’Osso et al., 2016), correlation studies have also linked ON to personality traits including perfectionism and narcissism (Oberle et al., 2017), psychological disorders such as a body dysmorphia (Bo et al., 2014), and obsessive compulsive disorder (Barrada & Roncero, 2018). Based on *Diagnostic and Statistical Manual of Mental Disorders* (5th ed.; DSM-5; American Psychiatric Association, 2013) criteria, ON is likely to be clinically categorized as avoidant/restrictive food intake disorder (ARFID; APA, 2013, p.334); however, there have been calls for its recognition as a distinct psychiatric diagnosis (Chaki et al., 2013; McGregor, 2017). Multiple orthorexia scales have been developed including the Bratman Orthorexia Test (Bundros et al., 2016), the ORTO-15 (Dunn & Bratman, 2016), and the Barcelona Orthorexia Scale (Bauer, Fusté, Andrés, & Saldaña, 2019), each attempting to define, predict, and assess risk factors for ON in different population groups.

### The Body and Society

Rather than framing them as personal dyscrasias, symptom complexes can be viewed as expressions of and outlets for overarching social and cultural phenomena. We live, Nicolosi (2006) argues, in an “orthorexic society” (p. 1), in which our symbolic relationship with food is now plagued with alimentary fears. This Nicolosi attributes to three main factors. The first is the weakening of the restraining power of traditional institutions (religion, ethnicity, community) on food intake and dietary practices. The human relationship to food, Nicolosi reminds us, is both material and symbolic. The “primitive” body was, in all likelihood, a communitarian body (p. 46) and a social field for alimentary expression. People in contemporary society frequently eat alone and many lack the traditional mores and rituals around eating practices which characterize more communitarian societies.

A second factor is the ever-increasing distance between the food producer and consumer. The present obsessive concern some people exhibit around their health and wellbeing exists within a world beset by food and ecological anxieties, themselves fueled by phenomena such as the global wave of obesity which government health programs seem powerless to prevent (Tremmel et al., 2017). A society in which the supply of food (particularly low-quality, subsidized; Guthman & DuPuis, 2006) is more or less unlimited has left consumers literally spoilt for choice, whereas the advent of modern storage, packaging, and labeling of foods now serves as a source of both reassurance and anxiety for customers. The third factor identified by Nicolosi is the framing of the body as an individual project (Nicolosi, 2006, p. 42). As western society has become more atomized, alimentary acts must assume new meanings if individuals are to retain any sense of influence and security around what they ingest. What Foucault (1998) called “technologies of the self,” that is, endeavors through which individuals seek to transform their own bodies, thoughts, and conduct, have governed human behavior for millennia. Twenty-first century society, however, has been characterized by unprecedented attempts to marketize self-care in the form body aesthetics as achieved through dietary and fitness regimes, such as low carbohydrate diets, bodybuilding, and more.

To Nicolosi’s 2006 list, we would add a fourth factor, which is the creation and marketization of multiple psycho-pathologies and subsequent “troubled person industries,” that is, industries, such as counseling, therapies and online forums, set up to bestow help and advice for those with problems (Gusfield, 1996). The term “medicalization” is a sociological concept that emerged in the 1960s to critique the expansion of medical terminology, interventions, and practitioners into those areas of the life that were formerly considered outside of the medical sphere (Mayes, 2014). As eating has become more medicalized, numerous institutions have emerged to supporting individuals with eating “problems.” Actual and emerging psychiatric categories then develop into widely accepted ideas and labels, which are deliberated in non-psychiatric contexts, on and offline, with mainstream and social media alternately glamorizing and pathologizing these trends to maximize consumer attention (Brechan & Kvalem, 2015). One example is the spate of “orexias” such as “bigorexia” (muscle dysmorphia) and “#bigorexia,”(Bratman, 2017) which now provide ongoing discussion material for twitter users, and potential clients for counselors, sports therapists, psychologists and with others working in troubled persons industries. Following this trend is a vast crowd of consumers, who for various reasons are vulnerable to both the messages conveyed about diet, health and fitness and their treatments, including those concerning extreme eating practices. In our article, we address the following questions: How do different actors (laypersons and professionals) position themselves around debates concerning health and eating on and offline, and what do their accounts tell us about tensions around the labeling of healthy eaters as “troubled persons”?

## Methods

### Overview

The aim of our study was to gather as many different perspectives on orthorexia nervosa, and the meanings ascribed to extreme “healthy” eating practices, as possible. We chose triangulation (investigator, method, and data triangulation) to capture distinctive dimensions, increase data richness, and converge different information on the same phenomenon (Carter et al., 2014). Three sets of on and offline qualitative data were gathered over a 6-month period by three different researchers working or studying in the same institution, under the direction of the second author. Our final data sets were the following: nine interviews with people self-identified as highly preoccupied with healthy eating (Cohort A: “Identifiers”); seven interviews with clinical psychologists, nutritionists, and a family therapist who have long-term experience of working with EDs (Cohort B: “Professionals”); and (3) “ortho” threads from an ED social networking site (Cohort C: “Posters”). All parts of the study were approved by the University Psychology Ethics Committee. The project ethics number is ETH17-18-1186.

### Recruitment

Cohort A, “Identifiers,” were recruited through poster advertising and social media, and later via snowball sampling. As there is no formal orthorexia diagnosis, the purpose of the study and its inclusion criteria (age 18 years or above and self-reported healthy eating that has taken over their lives) were described in lay terms to prospective participants. Other exclusion criteria of study were diagnosis of major psychiatric disorder, currently receiving inpatient treatment for an ED and inability to speak English. Place of residency and nationality were not stipulated. The final cohort of Identifiers consisted of six female and three male participants; seven were based in the United Kingdom and two in the United States (see supplemental material).

Recruitment of “Professionals” was purposive and aimed at those with diverse and in-depth expertise in EDs, and specifically ON. Our first point of contact was with organizations and charities working in the EDs field, who passed on our email to staff. We also posted details of the study on the EDs Professional Resource Network Facebook page. Cohort B finally consisted of three clinical psychologists, three registered dieticians, and one family therapist (also a qualified psychologist), who each worked regularly with clients with EDs in various medical and social care settings and private practices. All professionals identified as female, as attempts to recruit male participants were ultimately unsuccessful. Three professionals were based in the United Kingdom, three in the United States, and one worked in both countries. The table of participant interviews can be viewed in Table 1.

**Table 1.** Table of Participant Interviews.

Cohort	Participant Pseudonym	Gender	Area of Work/Occupation	Country of Residence	Interview Method
A	Jane	F	Student	UK	In person
A	Karen	F	Finance	UK	In person
A	Jake	M	Personal trainer	UK	In person
A	Tim	M	Finance	UK	In person
A	Jo	F	Physiotherapist	UK	In person
A	Edi	F	Homemaker	UK	In person
A	Stella	F	Student	USA	Phone
A	Clare	F	Professional	USA	Phone
A	Liam	M	Wellness coach	UK	Phone
B	Sue	F	Clinical psychologist	USA	Phone
B	Tina	F	Clinical psychologist	UK	In person
B	Anna	F	Registered dietician	USA	Phone
B	Nina	F	Clinical psychologist	USA/UK	In person
B	Wendy	F	Registered dietician	USA	Phone
B	Pippa	F	Clinical psychologist	UK	In person
B	Harriet	F	Family therapist	UK	Phone

After initial contact was made, participants were emailed a copy of the participant information sheet and consent form and given an opportunity to ask questions about the study. Interviews were arranged face-to-face or telephone/Skype according to the following participant preferences: Identifiers face-to-face (*n* = 6), telephone (*n* = 3), Professionals face-to-face (*n* = 3), and telephone/skype (*n* = 4). Interviews lasted between 23 and 46 min for people with ON and between 37 and 69 min for professionals. Participants were assured that the interview was private, and that any data used from the interview would be anonymized and would form part of a larger data set. Written consent was received prior to all interviews. Interviews were audio-recorded and transcribed verbatim by a professional transcriber who signed a confidentiality agreement.

### Data Collection

We designed separate interview guides with open-ended questions and prompts to direct conversations (Oliffe et al., 2019). Alison Fixsen is a registered complementary therapies practitioner, Anna Cheshire a research in health psychology, and Michelle Berry a registered dietician and psychology graduate. In the framing of our interview schedules, we drew both on our firsthand knowledge of clients across two continents (United Kingdom and United States) presenting with an ED and the wider literature on this topic. Both schedules were discussed and checked within the team. Interviews with lay people (“Identifiers”) explored the whole context of individuals’ eating choices and behaviors, including healthy eating choices; the reasons for healthy eating; influences on dietary choices; and the impact of their diet on daily activities, including physical and psychological well-being and social/educational/work life. Interviews with “Professionals” focused on their perception of and experience concerning fixations with healthy-eating, what they regarded as the key features of ON, how fixations with healthy-eating manifested in their clients, factors influencing these manifestations, and professional treatment strategies and their limitations. Following the interviews, a list of statements covering the key features of ON as uncovered in our analysis was sent to each participant, who was asked for feedback, and more specifically if the statements made sense, if they captured the experience of orthorexia, and if anything of relevance was missing.

In addition to analyzing face-to-face and Skype interviews, we analyzed data from threads selected from an “orthorexia” forum on an ED website aimed at supporting those attempting to recover from EDs, body dysmorphia, and obsession around weight and body image. Data were collected over a 2-month period between 2017 and 2018, and six threads selected over a 6-week period in 2019. For maximum variability, the data focused on threads that contained multiple responses from a variety of posters. Each thread contained between 10 and 68 comments. We followed the ethical guidelines advocated by Association of Internet Researchers (AOIR, 2019). The site we searched is accessible within the public domain, and the majority of forums and thread topics can be read without creating an account. All posts on the site are anonymous (the site does not allow users to use their real name as a username); however, to further protect privacy and anonymity, the site is not named in our article, and we have included only extracts from individual threads.

## Data Analysis

We used interpretive thematic analysis (Pope et al., 2007) to analyze the data, aiming at retention of an overview of participant stories as a whole. In the first stages of analysis, data from different interviews were considered separately, with notes made to summarize the basic outline of the core narrative and to identify key elements. By repeatedly reading transcripts of interviews and threads, the first author familiarized herself with the full data set. NVivo was then used to extract more codes and analyze different sections of the data in various ways. Close attention was paid to the actual language and phrases describing participant experiences. As ideas were generated, they were discussed with the research team. Data were coded using a modified constant comparison approach (Strauss & Corbin, 2015), inspecting and comparing all data and fragments arising in a given case and moving from a larger to more compact data set (Silverman, 2014) At different stages of data analysis, emerging codes and themes were discussed with the team. As final codes emerged, data were repeatedly rescanned manually to check for any missing or hidden codes or concepts.

## Findings

In this section, we turn to the narratives of healthy eating Identifiers, Posters on an ED forum, and Professionals in the ED field. We focus on the ways that different players interpret their own or others “healthy eating” practices and dietary choices, and their personal, social, and moral appraisals of such practices, starting with Identifiers. The figure summarizes the different themes, with an asterix indicating those themes also discussed by another cohort. We have used pseudonyms for all participants.

### Identifiers

#### Health benefits: “Food as medicine.”

Identifiers in our study ascribed to a variety of dietary practices for different reasons; however, all emphasized the significant health benefits of eating healthily and avoiding “junk food.” Treating food as form of medicine aligned with what they had learned concerning the benefits of eating in a biologically pure way, for example, “ This course was an introduction into how food can be used as medicine basically”; “It’s a preventative measure to eat well and to eat good produce now rather than loads of herbicides, pesticides, all that kind of stuff.” Some Identifiers had chosen to avoid particular foods for health reasons such as digestive issues, skin problems, or menstrual pains. As Tim explained, refraining from low-quality foods could go a long way toward prevent future illness:

Now that I’m getting older you have to start worrying about things like cancer . . . Parkinson’s, Alzheimer’s, all that type of stuff, and a part of why you eat healthy is to try and avoid that in a lot of ways. I think there’s a lot of evidence that cancer can be linked to an unhealthy diet and eating high fat fried foods—who knows what that does in terms of the risk of getting cancer? (Tim)

#### Moral issues: “Meat grossed me out.”

Over time, what and how they chose to eat had become one of the ways in which Identifiers defined themselves, for example, “I’m a pescatarian”; “I’m guess I eat mostly a paleo-ish [diet].” Several of the cohort had chosen to abstain from meat for ethical reasons: “Not eating meat is not a dietary thing per se, it’s animals,”; “I started cooking for myself and meat grossed me out, the blood and bones.” Stella had been influenced in this respect by her husband, who had written a meat-free cookbook. After watching ecological and animal rights documentaries, Jane was even more convinced that “going” vegan had been the right choice: “‘Cowspiracy’ . . . It’s mainly about how the general meat and dairy industry is bad for the environment.” There were a few downsides to being vegan, such as having to plan more when choosing where to eat out or making people aware of one’s dietary requirements, but there was also the exhilaration of being part of a growing culinary trend: “[It’s] exciting to figure out what vegan restaurant in London haven’t I been to yet and going to try them out”(Jane).

### Achieving the Ideal Body

For some (but not all) Identifiers, healthy eating with part of their chosen lifestyle, in which food was used to promote fitness levels and a particular body shape, for recreational or business purposes. Three of the cohort trained in bodybuilding and used dietary manipulation and supplements as a way of achieving their ideal body shape and muscle tone goals:

It’s a good time we’re speaking, to be fair, because I’m about to do a competition in the next seven weeks. So . . . I’m on like a, a strict diet, and right now it’ll be more like six meals a day, spread out between maybe two and a half to three hours. And then meals will be just kind of intake a high protein, a low carb, but healthy standard fats. (Jake)

Sticking rigidly to his diet before a competition was not something that Jake actually liked; it was “a really big mental thing,” as he genuinely loved food. However, his partner and personal trainer, along with motivation videos on YouTube and Netflix, helped to keep him on track: “It’s good to watch people like that, how they push through when you’re struggling.” Body builder Liam had “done a lot of research into nutrition from the body building, power-lifting side.” Initially he had been “heavily influenced” by names “in the industry” who post on Twitter and YouTube, “but then you start to develop more of your own understanding” of nutrition. Now working to establish his own online business, Liam believed that his good physical shape would work in his favor: “The [online] visuals draw you in . . . probably the biggest element is physical appearance.”

### Influence of Family

Jo and Stella also spoke of on-going concerns about body shape but related these to a personal or family history of EDs. Jo, who used purging, “as a bit of a crutch,” found her on-going inner dialogue about food and body weight a real trial; “At this point in my life where there’s like multiple layers of shit, I’m like ‘shit, this, this is stressful weighing and counting food!’” Stella had a mum who had bulimia, whereas her grandmother (a “yo-yo dieter”) had been on numerous slimming pills and dieting programs: “I look at my mum and grandma and I say, ‘I don’t want to be like them’.” Despite her attempts to rationalize such behaviors, for Stella, overeating continued to provoke feelings of disgust within herself and toward others:

I don’t know if it’s because when, when I quit swimming, I went from 60 kg to 82 fucking kilos, right, I was disgusted in myself. So now I have an even more skewed opinion of fat people, I think it’s disgusting. (Stella)

For other Identifiers, childhood evoked very different food memories. Edi, one of the older members of the cohort, described herself as a little over-weight but “rather obsessive about healthy eating.” Having grown up in Italy, good dietary habits had been instilled into her at an early age: “there was always a delicious meal on the table, we all sat down at the table together as a family.” Liam also cited his mother as being the biggest influence on his dietary interest and choices: “All her meals are very, very good in the traditional healthy eating landscape.”

### Adherence to a Regime

Participants in this study were requested to talk at length about their dietary preferences and eating schedules and most described them in detail. Although the planning and monitoring of a “healthy” diet regime was undoubtedly preoccupying, even sometimes stressful, it provided you with “reference points to work from.” Identifiers experienced feelings of disappointment or guilt after having deviated from their diet, for example, Tim might decide, after “indulging” in a croissant, to skip the next meal. Liam admitted that he felt worse “mentally” if he did not eat healthily for some time, “because I feel like I am not developing toward my goals.” Stella spoke of the internal diatribe that might go on in her mind when faced with dietary temptations:

If I were like, “Oh I can’t go get an ice-cream because I just had one yesterday or last week . . . then it starts up mental monkeys, of ‘this is bad, I’m going to feel bad, I’m going to hurt, I’m going to get out of control and eat all the ice-cream in the universe’. That kind of thing.” (Stella)

Most Identifiers could recall social scenarios in which adhering to their usual dietary choices had proved awkward or impossible, nevertheless social isolation (a frequently cited negative consequence of ON) failed to emerge as a major issue for the full cohort. On the contrary, personal contacts with people who followed or advocated similar dietary practices (partners, trainers, therapists, friends), along with information they had gathered from nutritional literature and media, could help to reinforce personal beliefs and regimes. For example, Jake had a dietician wife who advised him on nutrition, whereas Clare had lots of vegetarian friends “who had impressed me.” In summary, though Identifiers were aware of the personal and social costs of following a relatively strict diet, all were convinced that of its benefits and most said they felt much better for it. As Clare explained,

I like the way I eat. I think it’s a healthy way to eat, I think, I feel comfortable . . . and ultimately, I don’t see myself changing my diet . . . And I think health wise I’m doing well. I’m 61 years old, I’m going, almost 62, my last checkup was great, I feel good, I don’t have to take a lot of medications and ultimately, proof’s in the pudding. (Clare)

### Posters

#### Body anxieties

The topics of weight and body image were frequently raised on the ED forum. On a thread, “what are some of the things you obsess over?,” checking calories, losing weight, and maintaining a certain image were priorities: “I only eat healthy foods since you can’t get fat from them.” Posters, like Identifiers, had strict rules around food and eating, but were more self-berating and more concerned about their ability to maintain these rules and the consequences of deviating from them: “I have so many rules and fears”; “Kept making them [rules] stricter and stricter.” Following their restrictive regimes did not however rid Posters of anxieties around the effects of eating forbidden foods: “I can’t let go of my anxiety . . . I live a sad and pathetic existence.”

#### “Cycling.”

Online users adopted their own argot to describe their common experiences. What was known as “cycling” (alternating between orthorexia eating and a former ED) was also commonly discussed on the site. Posters on the site wrote of how orthorexia eating had replaced a past ED, for example, “I suffered from anorexia before it developed into ortho (orthorexia) . . . I guess now I’d be called a restrictive anorexic.” For some, this was seen as a sign of recovery; “I developed ON [to] counteract all the damage I’ve done.” The cycle of eating and purging (self-induced vomiting and/or laxative use) was a frequently discussed topic, with which many battled:

I didn’t realize it but as i started to add more healthy calories into my diet, i added more unhealthy ones too & i began to binge at night. now i have put on 20ish pounds & even though i look healthy, i miss that control of only eating cleanly. And now when i have those binges of unhealthy things, i have resorted to purging.

Breaking one’s resolve was a source of distress, but to keep “clean” and healthy, purging was seen as sometimes necessary: “If I don’t eat clean, I will get sick.” Warnings were issued about the dangers of falling back into old patterns while attempting to clean up or lose weight, and advice offered to those who might feel inclined to purge after “messing up,” for example, “Purging sets a person up to binge and purge. How do I know this? Been there, done that. It’s hell.”

#### “Fucked up.”

A dilemma for Posters was that, while “ortho” in the sense of “healthy eating” had to be a good thing (e.g., “I honestly believe being orthorexic isn’t that bad . . . I’m eating whole plant foods and not eating donuts, pizza and crap”), being obsessed to the point of complete irrationality was very distressing, for example, “i pretty much just want to die whenever i feel somewhat pressured to eat ‘normally’.” Harsh language was used to describe the sense of personal disgust Posters felt when they transgressed from their self-imposed regime (e.g., “failure,” “pathetic,” “fucked up”). Like Identifiers, some Posters blamed their families for their extreme attitudes to food, for example, “Mother and sister would guilt me for like eating a bag of chips or something ridiculous”; “eating decisions are governed by a single rule, ruthlessly drilled into my head by my retired gymnast parents.” Being with friends and family was widely regarded as fraught with alimentary dangers, for example, “I’m tired of being upset about all the nasty food my mom makes me sit down and eat against my will.” In contrast, the website was portrayed as a safe place to express one’s fears and emotions. Sharing their transgressions and concerns was, for some Posters, a huge relief, “Realising that I’m not alone. That it isn’t just a failure on my part but a biological response.” Messages of support and reassurance were offered to others who had broken rules; for example, “The holidays is *so* rough to eat through with disordered food stuffs, I commend you for doing it.” There was also a celebratory tone in some threads concerning a shared adherence to food purity:

I must be really demented because I don’t see how this is sad. I mean, it’s fabulous. Our binges are all about healthy foods. That’s a win.

## Professionals

### Orthorexic Society

The idiom, “orthorexic society,” has been used to critique the way in which food and health have become inextricably linked, to the point where this becomes the central organizing determinant guiding contemporary food selection (Nicolosi, 2006). Professionals in our study, while not describing society in quite these terms, voiced strident opinions concerning attitudes to food, health and appearance in society, and their effects on young females in particular. Sue, who had worked with EDs for over three decades, spoke of “witnessing the emergence of idiosyncratic, irrational ideas about the power of food to affect health and wellbeing.” Our environment had, Anna considered, “provided us with lots of rules about how to get it right, and certainly for a woman body image, food and eating, is the kind of big, dominant story.” There were feminist issues here; when Nina moved to Los Angeles (LA), she was struck by how objectified women there were; “so much was about body size, and also a sense of inferiority.” Until recently, it was her higher income clients that leaned toward orthorexia; however, she had noted a restricted, elitist type of eating emerging within the U.S. Latino community, as part of an ethos of “finding a better life.” Wendy spoke of a growing panic over the health crisis in the States, with major food companies competing with each other over this: “Let’s make sugar look bad, let’s make dairy look bad.” These messages were prominent everywhere in LA; “There’s billboards with, ‘oh let’s freeze off your fat’, or ‘try this juice cleanse’.” Although LA might be seen as the “home of orthorexia,” Professionals had noted very similar trends in other countries. Pippa, who had worked with sports people from around the world, believed the high prevalence of orthorexia in her male clients had a lot to do with the pressures of that particular work environment, but also the influence of social media on attitudes toward food and body image:

[ON] almost seems more of an acceptable illness for a male to have- whereas there’s a lot of stigmas, I think, with anorexia in males generally, so people don’t always admit it. And I think a lot of that [pressure] comes from the bodybuilding community . . . and Instagram . . . food blogs. (Pippa)

### Parallels With Other Disorders

Professionals as a body considered ON to be associated with anorexia and bulimia nervosa, with many clients sharing the obsessive, perfectionist traits of anorexics: “Neat and tidy. Conscientious. High achievers in sports. They’ve got healthy eating and they go to the local grammar school, that’s another flag.” (Harriet). Unlike anorexia nervosa and bulimia, however, “healthy eating”/restrictive eating was “so validated . . . rewarded in our society.” You had to point out to clients their “underlying fear [around food] and call them in on that.” In some cases, orthorexia was used as an “escape route” from the label of anorexia; “So people stop being anorexic in the classical sense and they they’ll evolve into, ‘well I’m totally cured but I just don’t eat dairy, bread, gluten [etc.]’” (Sue). Most clients whom dietician Wendy considered as “orthorexic” had started out with digestive problems or food intolerances, and then their diet became “more and more restrictive,” to the point where “they cut out everything.” Sue was dismissive of many so-called food intolerances; “It’s not quite summarizing, but a kind of misattribution of a normal, healthy process—they will take normal, healthy digestion and pathologies it.” Tina had also noted a trend in obsessive eating patterns among young men with low self-esteem (“you might call them muscle dysmorphic” she added) who attended her London clinic:

He [her client] was preoccupied by the fact that certainly among the gay scene . . . it was just commonplace for guys to walk around with their shirts off and it was all about the physicality . . . And he would just sit there and say, “I know it doesn’t make any sense.” (Tina)

### “Murder to Treat”

Trying to get an orthorexic patient to move out of their “orthorexic” pattern was described as “absolute murder,” because it was “a safety behavior” and “a defense against fear and anxiety,” similar to people with obsessive-compulsive disorder (OCD) who wash their hands compulsively. There was also the moral superiority associated healthy eating practices that professionals had to contend with:

With vegetarians and vegans you get this added layer and you might ask . . . “ do people with orthorexia come and ask for help?” and the answer is, they’re exactly the same as people with anorexia . . . no one comes for treatment for orthorexia and says “I’m orthorexic, I want to change,” because in their view that would mean eating the stuff which disgusts them. (Sue)

Speaking from her experiences in California, Anna described a “very interesting trend” in which the entire family could hold these core beliefs about food and health, although most of the professionals regarded the mother’s role as central; “Majority wise it’s the women or the mother, the matriarch figure, running the ideology and running the thought process of the orthorexic tendencies.” Sue blamed the parents for setting these eating trends and sending out unhelpful messages to their children; “Parents think it’s smart and cool, ‘oh I’m not eating carbs, oh I’m not eating meat, our family is becoming vegetarian’, they’re not wise.” Family therapist Harriet felt this went right to the root of parenting in modern society:

Homelife, it’s something about not being nurtured . . . you know, mums and dads aren’t available, either they’re working long hours . . . there’s some very strict families, and that’s the only thing they’ve [the young person has] got in control of themselves—how much they put inside their bodies. (Harriet)

Professionals came up with their own treatment strategies for orthorexic clients. Anna would sometimes take these clients out for an ice cream: “I mean it’s much easier if it’s like, we’re going to do it [eating ice-cream] together and then we can talk about it after.” Nina would try to encourage a more playful attitude to food and eating, and the use of neutral language rather than derogatory terms such as “junk food.”

### “Medicalizing” Restricted Eating

Professionals were divided in their opinions about the usefulness of the term “orthorexia nervosa.” Pippa was part of a working group on orthorexia, who were keen to get ON included in the DSM. Nina, on the other hand, was concerned that, were it to become an official diagnosis, it could be used in schools to single out picky eaters such as her child with Asperger’s syndrome. Sue saw no virtue in pathologizing healthy eating unless it was causing gross nutritional deficiencies or creating excessive paranoia about food. Having recovered from a short-lived ED herself, Sue considered expanding the DSM to include clinically nonsignificant symptoms and behaviors to be a dangerous trend. Where treatment had improved could be seen in “how we understand such patterns, and how we think of them in terms of a person’s ability to function.”

## Discussion

**Figure 1.**
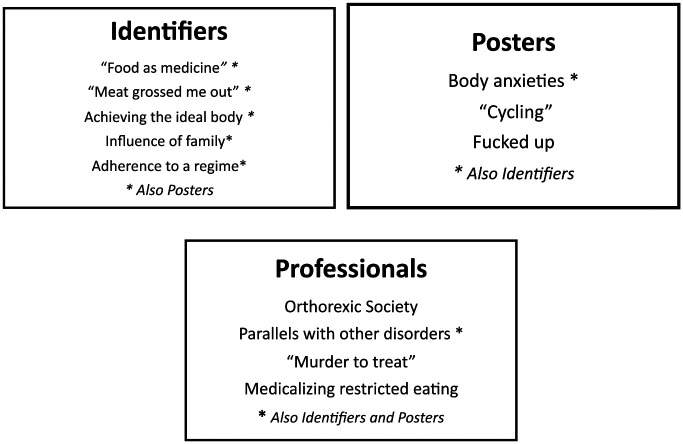
Comparison of themes from the three cohorts.

We have presented the perspectives of three cohorts with different and divergent interests in extreme healthy eating practices and summarized the themes in Figure 1. What we term “orthorexic practices” (to distinguish this term from a clinical definition of ON) emerged as highly complex phenomena. Depending on their personal and professional positions, participants made sense of and rationalized their attitudes and feelings concerning healthy eating and aligned themselves accordingly. For Identifiers and Posters, narratives around obsessively healthy eating took place in multiple contexts, including online. Life for these cohorts revolved, to a greater or lesser extent, around food choices and food preparation. Although aware of the personal and social costs of following a more or less strict diet, being selective in one’s eating practices was, for Identifiers in particular, framed as a form of self-care technology which at the same time enabled them to identify as being part of a wider cultural or social movement, such as veganism, clean eating (Allen et al., 2018), or body training (Håman et al., 2017).

Body dysmorphic disorder (BDD)—described as a distressing or impairing preoccupation with imagined defects in appearance (Phillips, 1991)—is considered characteristic of much-disordered eating (Bo et al., 2014). Although some Identifiers’ body appearance spoke of having an ED, posters in this study were more overtly preoccupied with physical appearance, in particular personal thinness and weight control. Norms and rules apply to these types of online realms (Oksanen et al., 2015), including the censoring of comments which might encourage self-destructive thought patterns and behaviors (Fixsen & Ridge, 2017). The tone of the threads we searched was more self-deprecating than the responses of Identifiers, who were more inclined to frame their dietary discipline as part of a rationalized, sometimes ethically motivated lifestyle choice. Anonymity on the website may have made it easier for posters to “confess” to feelings of self-disgust following dietary transgressions, convey their need for peer support and express their views about others with EDs. As in other studies of online communities, Posters employed in-group language and metaphors (author) using terms such as “ortho” (orthorexia), “b/p” (binging and purging), “cycling” (between EDs), and “messing up” both to identify and categorize their experience and allow for sharing of emotive (Mizes & Arbitell, 1991) and stigmatized topics (Fixsen & Ridge, 2017; Oksanen et al., 2015). Despite their different positions on or off any ED scale, Posters and Identifiers shared a common interest in the pursuit of a better, “cleaner” self, whether ethical (e.g., associated with animal welfare) or aesthetic (e.g., associated with physical appearance and social media image).

Professionals, some of whom had personal investment in promoting healthy eating, voiced strong disapproval of society’s obsession with healthy eating and for the parents of clients who appeared to take these messages too far. Their role was to challenge and modify obsessive patterns before any real psychological or physical damage was done. Orthorexic eating was seen as “murder to treat” due to the high validation of some manifestations of ON in society (e.g., healthy eating, weight loss) and its multifactorial nature, including perfectionist tendencies, health and body anxieties, fitness regimes, past trauma and social media. Having summarized our findings, we now discuss our interpretation of the social construction of orthorexia from the perspective of morality narratives around food and self, and the psycho-politics which underpin ON.

### Orthorexia: Morality Narratives

The prevailing view of disordered eating practices is that they are particularly difficult to treat, as people usually deny that they have a problem and are therefore resistant to seeking help or treatment (Musolino et al., 2015; Vitousek et al., 1998). Those with orthorexia can be seen as vulnerable persons faced with constant health and fitness advice circulating through a society obsessed with the pursuit for the perfect body (Widdows, 2018) in which the achievement of health is a moral virtue to which citizens of all ages must aspire (Pond et al., 2010). Among our sample of Identifiers and Posters, there are those for whom both these statements might be applied. Patients with disordered eating, Musolino et al (2015) suggest, resist therapy by hiding their practices, instead seeking safety through their own form of care and around culturally normative ideals around food and bodies. In a somewhat similar vein, professionals in our study recognized ON as being a safety behavior in a self-obsessed society, but still one that was “murder” to treat. This may help to explain the censure expressed by some professionals toward not only their clients’ resistance behavior but also the parents and communities who acted as collusive agents. How then does this fit with the viewpoint of the healthy eater/ON sufferer themselves? Is it simply a case of different perspectives or a clash of values concerning individual and social responsibilities? Is it the moral responsibility of clinicians and researchers to identify “orthorexic” individuals for help seeking? (Moller et al., 2019; Musolino et al., 2015). We would suggest that society and its representatives (clinicians, media, researchers) wrestle with the dilemma of not knowing whether to vilify or glamorize, reject or adopt a term which sits between a life promoting philosophy and behavior, and a deviant or even subversive one. If the tension between how far we go in order to defend our bodies and postpone our own demise is seen as a humankind rather than an individual concern, then orthorexia could be framed as an internalized debate which, within our self-obsessed society, any one might struggle with (Nicolosi, 2006).

### The Labelling of Healthy Eating

As Gusfield (1996) asserts, the cultural concept of a social problem is not separate from the social institutions which deal with these problems. Medical diagnoses are negotiated creations that emerge from and shape social and moral contexts, expressing and defining what is “normal” and what needs to be treated, and rearranging individual and professional identities in the process (Jutel, 2014). As eating has become more medicalized, numerous institutions have appeared to provide services and support for individuals with eating “problems.” What Cohen (2015) calls “psy-professional” actors, that is clinical psychologists, dieticians, sports therapists and others with investments and interests within these fields, seek to employ their specific definitions of reality and create better adjusted individuals (Cohen, 2015; Wood-Barcalow et al., 2010). The trend in self-diagnosis and the multiplication of “troubled persons” (Gusfield, 1989) agencies and forums on the Internet further suggests the need to reconsider both the institutions influencing diagnoses and the processes leading to “issues” in the first instance. At the same time, the power to ultimately define and treat mental illness through the modification and addition of categorizations in each new DSM edition remains the prerogative of psychiatry (APA, 2013; Cohen, 2015). Some “psy-professionals” may disagree with particular categorizations, yet face increasing pressure to adopt such labels for financial, validity, and insurance reasons (Greenberg, 2013; Jutel, 2014).

We suggest that as socially constructed disorders such as ON find their way into the DSM several things occur. First, as clinically defined conditions they acquire stigma (Nevin & Vartanian, 2017) and circumscribed identity. Second, their exploitability and value within the global marketplace increases. Third, in order to receive or give support, people and practitioners (medical and nonmedical) feel the need to adopt the language and labels of the disorder. This leads us to question in whose interests it lies for ON to be included in the DSM and to advise caution on the part of those seeking its recognition. Our findings suggest that while some individuals on the healthy eating spectrum might well be in need of help and support, there are dangers in overmedicalizing what may for others be largely a preferred lifestyle. With discourses on ON becoming common parlance in professional and commercial settings (Horwitz, 2012), there is a risk of some social institutions “feeding” the eating problems they purport to address.

### Strengths and Limitations of Our Study

Headed by the Anna Cheshire, the team was tasked with investigating and mapping the defining features of ON and the various influences on its development. Although we shared this common aim, we brought to the project our diverse, multicultural experiences as health practitioners and researchers, and understandings from psychology, health sciences, and complementary therapies. Our combined analysis involved lengthy discussions about our findings, the existence or not of ON as a disorder and our concerns that, given our personal interests in healthy foods, we could have strayed into territory which tested our personal comfort and beliefs. What we have presented in this article is a constructionist interpretation of healthy eating/orthorexic practices and their psychosocial roots, from different perspectives. Although our methods might be considered ambitious, exploring a range of perspectives together has allowed us to interrogate the social construction of ON more thoroughly and to present some potentially unique findings. Finally, we acknowledge the limitations of this study in terms of its size and distribution. Although we have chosen to study ON across continents in this way, there may be contextual factors between countries that are worth exploring. Our dip into the social world of online communities suggests this is an important area of research and we welcome further critical research in this area.

## Supplemental Material

ON_SSI_guides_ – Supplemental material for The Social Construction of a Concept—Orthorexia Nervosa: Morality Narratives and Psycho-Politics Click here for additional data file. Supplemental material, ON_SSI_guides_ for The Social Construction of a Concept—Orthorexia Nervosa: Morality Narratives and Psycho-Politics by Alison Fixsen, Anna Cheshire and Michelle Berry in Qualitative Health Research
