# Supplementary material for: The Social Construction of a Concept—Orthorexia Nervosa: Morality Narratives and Psycho-Politics
Source: Qual Health Res. 2020 May 18;30(7):1101–13. doi: 10.1177/1049732320911364 (PMC7411527; doi:10.1177/1049732320911364)
Supplement: ON_SSI_guides_ – Supplemental material for The Social Construction of a Concept—Orthorexia Nervosa: Morality Narratives and Psycho-Politics [file ON_SSI_guides_.pdf]

ON study: Semi-structured Interview guide

Introduction: Briefly explain study to each participant

Cohort A Questions :

Can you tell me about your eating habits?

  a. Any food / food groups you include?
  b. Any food / food groups you avoid?
     i. Can you tell me reasons that you avoid foods / food groups?
- Can you tell me about your daily routine?
  a. Can you describe your food preparation?
  b. Can you describe your eating schedule?
- Can you tell me how you chose your current diet?
- Can you tell me about any influences on what you eat?
  a. Can you tell me about anything you have read / watched?
  b. Can you tell me about any influences from social media / friends and family / job?
- Can you describe what you find positive about how you eat?
- Can you tell me about any negative impacts your diet has had on your life?
  a. Can you describe any effects on your social life?
  b. Can you describe any effects on your well-being?
  c. Can you tell me about your stress levels?
     i. How does your diet affect stress?
- Can you tell me about any impact on your physical well-being? E.g. energy levels, skin issues
  a. Can you tell me about any effects upon your finances?
  b. Can you tell me about any effects upon your job / education (if student)?
- Can you tell me how it feels when you don't follow your diet or eat unhealthy food?

Cohort B Questions :
- Can you tell me a little about your background?
- What is your experience of working with individuals with ON?
- What do you see are the key features of ON?
- How is ON expressed or manifested in different people?
- What effect does it have on individuals
  a. psychologically b. physically c. socially, education/work d. financially?
- What do you consider is the tipping point between healthy eating and ON?
- What do you think influences the development of this eating disorder in individuals?
- How would you consider it relates to other eating disorders, particularly anorexia?
- What if any cultural differences or socioeconomic differences have you seen in individuals with ON?
- What has been your experiences working therapeutically with this group?
- Any further comments/things they would like to add?
